# Students home alone—profiles of internal and external conditions associated with mathematics learning from home

**DOI:** 10.1007/s10212-021-00590-w

**Published:** 2022-01-05

**Authors:** Sarah I. Hofer, Frank Reinhold, Marco Koch

**Affiliations:** 1 ETH Zurich, Weinbergstrasse 43, 8092 Zurich, Switzerland; 2 Institute for Mathematics Education, University of Education Freiburg, Freiburg, Germany; 3 Individual Differences & Psychodiagnostics, Saarland University, Saarbrücken, Germany

**Keywords:** Home schooling, Profile analysis, Motivation, Socioeconomic status, Mathematics education, COVID-19

## Abstract

This study aims at describing differences in internal and external resources of students to handle mathematics learning from home. Based on data from *N* = 223 7th-grade secondary school students gathered via an online survey at the end of the first school year during the COVID-19 pandemic, we used latent profile analysis to identify student profiles defined by the internal factors *perceived value* and *success of students’ math learning from home* and the external factors *family support* and *teacher support*—all specifically related to home learning. A number of general learning conditions, comprising internal (e.g., sustained attention) and external factors (e.g., socioeconomic status), are included as outcome variables. The best-fitting four-profile solution suggests one profile with comparably unfavorable internal and external resources. About 35% of the students are assigned to that profile. The other three profiles show combinations of, relative to the sample, more and less promising specific home learning and general learning conditions suggesting that these students have different resources available in the face of learning mathematics from home.

**Supplementary Information:**

The online version contains supplementary material available at 10.1007/s10212-021-00590-w.

## Introduction

The data for this study was collected during the COVID-19 pandemic in the summer of 2020. Schools closed globally for several weeks. Although learning for school at home is an integral part of many educational systems, having the whole student body of countries all around the world learn half of the time or even *only* at home for a prolonged time is a situation unparalleled in modern history. Factors associated with learning from home become visible in these times more than ever before—as seen through a magnifying glass. The data gathered in this exceptional time can inform us about internal and external conditions of more or less successful learning at home, which is of high relevance, even beyond the current crisis.

**Fig. 1:**
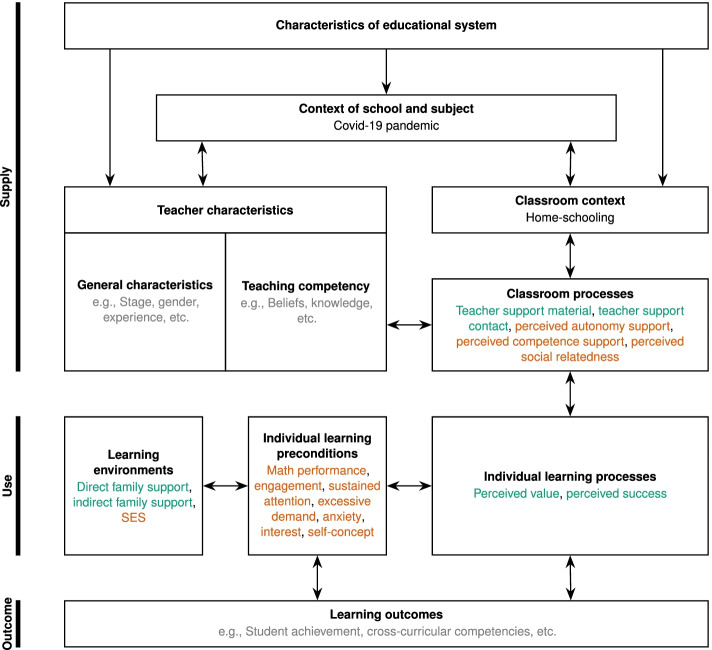
Adapted Supply-Use-Model based on Brühwiler and Blatchford (2011). Variables used in this study are printed in green (specific home learning conditions) and orange (general learning conditions). The internal factors implemented in this study are represented by “individual learning preconditions” and “individual learning processes,” whereas external factors are represented by “classroom processes” and “learning environments.”

On a superordinate level, the success of instruction at school can be expected to depend on two factors: supply (the available learning opportunities) and use (whether and to what extent a student makes use of the learning opportunities provided). The corresponding model is often referred to as the Supply-Use-Model (Brühwiler & Blatchford, 2011; Helmke & Weinert, 1997). Figure 1 shows an adapted version of this model. The variables used in this study are embedded into this model and highlighted in color. In addition to the distinction of variables in the existing model, we differentiate between *specific home learning conditions* (printed in green), comprising variables that very specifically relate to the conditions of learning from home, and *general learning conditions* (printed in orange), comprising variables that have consistently been found to be associated with learning on a more general level. Our adapted Supply-Use-Model can hence be used as a guiding framework to embed and connect relevant variables describing how different students make use of different learning opportunities taking into account the exceptional situation of learning from home during the COVID-19 pandemic.

To investigate potential effects of school closure and learning from home due to the COVID-19 pandemic, Schult et al. (2021) compared the 2020 cohort (school closure) of German fifth-graders to the cohorts of 2017–2019 (regular classroom practice). They could show a decrease in math competencies for the two lowest quartiles of students regarding socio-cultural capital for the 2020 cohort—yet, an increase for the highest quartile. In a comparable study by Engzell et al. (2021), estimations for learning losses due to the pandemic situation were higher for children within the lowest two tertiles in terms of their parents’ educational level than for children in the highest tertile. These comprehensive studies underline the risk of certain groups of students to suffer considerably from school closure and learning from home during the pandemic. However, existing research on that topic so far did not apply a person-centered approach that allows analyzing systematically occurring combinations of variables (i.e., internal and external conditions) to describe different student profiles of internal and external resources to handle learning from home. A nuanced description of students’ resources for learning from home can inform specific interventions and follow-up research.

Accordingly, in this study, we aim at identifying profiles characterized by students’ perceived success and value of learning from home and their families’ as well as their teachers’ support during this time (*specific home learning conditions*; see Fig. 1). In order to get an idea of potential explanatory variables, we relate these profiles that are based on internal and external factors directly associated with the specific conditions for learning from home to more *general learning conditions* such as the student’s cognitive and motivational-affective prerequisites and the family’s socioeconomic status (SES; Fig. 1). Both within *specific home learning conditions* and *general learning conditions*, we hence distinguish between internal and external factors that are expected to contribute to students’ success in learning mathematics from home. While internal factors relate to students’ attitudes and underlying motivational, affective, and cognitive prerequisites, external factors are located outside the student and refer to supportive (or obstructive) conditions provided by their teachers and families.

Because of considerable differences in teaching, learning, and motivational and emotional orientations towards different school subjects, we focus on mathematics as one core subject in this study. Mathematics proficiency is essential for educational success in different subjects and can be conceived of as a gatekeeper to many career paths (Jones, 2014; Stone et al., 2008). Yet, quite consistently over the last decade, about 20% of the 15-year-olds in the PISA survey fail to achieve the minimum mathematical proficiency that is considered a prerequisite for full participation in society (National Center for Education Statistics 2017; OECD, 2016, 2019). Therefore, it is of specific interest to identify students that should be considered at risk to lose ground when learning mathematics from home.

Among the specific home learning conditions, teacher support during learning from home may be one important external factor influencing how well students handle remote schooling. There is already evidence that the guidance and structure provided by teachers outside the regular school context varies considerably (Eickelmann & Drossel, 2020; Ortiz, 2020). In addition to teachers’ provision of material and guidance during remote learning, the home learning environment—i.e., external support from the students’ social environment—might, in parts, compensate for the lack of structure and assistance during remote schooling (Coley et al., 2020). Factors internal to the student have to be considered as well when describing resources available in the face of learning mathematics from home. Students’ motivation to make use of remote learning opportunities can be conceived of as the value students attach to learning math from home and its perceived success (Eccles & Wigfield, 2002; Eccles et al., 1983; Vroom, 1964).

These internal and external factors that directly reflect students’ experiences during remote schooling (*specific home learning conditions*) can be connected to more general internal and external conditions related to mathematics instruction and learning (*general learning conditions*). Among those conditions are internal factors such as students’ engagement or interest, and self-concept regarding mathematics (e.g., Broadbent, 2017; Broadbent & Poon, 2015; Fischer et al., 2020), as well as the family’s SES (e.g., Coley et al., 2020). In the next sections, we provide further details on the *specific home learning conditions* and *general learning conditions* considered in this study to investigate systematic differences in the resources that are available to the students when learning mathematics from home.

### Specific home learning conditions

#### Internal factors: expectancy and value

According to expectancy-value theory (Eccles & Wigfield, 2002; Eccles et al., 1983; Vroom, 1964), domain-specific subjective task value—Do I want to do it?—and perceived success–Can I do it?–explain the motivation to engage in an activity, i.e., the energy and attention directed towards a task. The result of a student’s contemplation of these two questions regarding remote schooling can be expected to influence the success of their endeavor to learn from home. This theory offers a widely accepted theoretical foundation for explaining students’ motivation to engage in learning—following the overarching idea that students’ achievement motivation, perseverance, and choice of tasks are directly related to their expectations of success and the subjective value they assign to the task (e.g., Kosovich et al., 2017). In the context of learning mathematics from home, we understand subjective task value as students’ perceived value of continuing studying mathematics even when schools are closed and making use of the learning opportunities provided by their teachers, i.e., video lectures, digital or non-digital learning tasks, or other educational material to build up new or deepen already existing mathematical knowledge. Expectation of success indicates how students anticipate and evaluate their success in learning mathematics from home. Both aspects can be expected to represent seminal drivers of students’ motivation to keep learning during the pandemic situation, in particular, since they have been closely linked to successful self-regulated learning (Artino & Stephens, 2006; Bergey et al., 2018; Panadero & Alonso Tapia, 2014).

#### External factors: family and teacher support when learning from home

While the link between family environment and school success is well-documented for regular school contexts (Elliott & Bachman, 2018; Foster et al., 2005; Froiland, 2021; Müller & Ehmke 2013), it can be assumed that aspects such as parental support in doing homework (Almutairi, 2021; Gonida & Cortina, 2014; Zady & Portes, 2001) and having a distraction-free workplace at one’s disposal (Baticulon et al., 2021; Soria et al., 2020) may increase in relevance when shifting from in-school mathematics learning to learning mathematics from home (see also Köller et al., 2020). Data gathered during the COVID-19 lockdown in England (Andrew et al., 2020) shows that access to resources such as computers and a dedicated study place at home is positively associated with learning time. Access to IT infrastructure might be more or less seminal for successful remote schooling depending on the teachers’ reliance on digital resources. A safe place to study, however, can be considered a universal precondition for efficient learning from home.

In addition, we know for some time now that there is a strong link between teacher behavior and students’ engagement and learning during regular classroom instruction (e.g., Hiebert & Grouws, 2007; Skinner & Belmont, 1993). During remote schooling and online teaching, there is evidence for considerable differences in the quality and quantity of learning activities initiated by teachers (e.g., Lohr et al., 2021; Wößmann et al., 2020). Based on data from three German-speaking countries collected during the COVID-19 pandemic, Huber and Helm (2020) emphasize the role of teaching quality during remote schooling over and above home resources. Establishing or maintaining close contact including regular and timely feedback seems to be particularly important to help students learn on their own (Bansak & Starr, 2021; Hofer et al., 2021; Köller et al., 2020). Providing material and structures (close contact) that allow students to continue learning mathematics while school is closed can be regarded as a crucial resource.

### General learning conditions

#### Internal factors: cognitive, motivational, and affective prerequisites

In the following section, we briefly introduce central cognitive (sustained attention and mathematics performance) and motivational-affective factors (engagement, excessive demand, anxiety, self-concept, and interest) that can be expected to play a role in mathematics learning from home. To start with, sustained attention is a cognitive process that allows learners to persistently focus on a task and maintain effort over extended periods of time (Blotenberg & Schmidt-Atzert, 2019). Learning involves complex processing of information that requires sustained attention (Oakes et al., 2002; Schweizer & Moosbrugger, 2004). In line with this argument, sustained attention has been shown to be related to school grades in mathematics and German (Steinmayr et al., 2010) and, in particular, to homework completion (Axelrod et al., 2009; Bryan et al., 2001). To manage working for school at home, students have to stay on-task and resist distractions. Sustained attention can hence be considered an important prerequisite for learning mathematics from home—which demands higher self-regulation skills from the students than regular classroom instruction to maintain engagement (e.g., Broadbent, 2017; Broadbent & Poon, 2015; Fischer et al., 2020). As a second cognitive factor, we consider domain-specific prior knowledge as one of the strongest predictors of learning in general (e.g., Dochy, 1994; Maguire et al., 1999; Schwartz et al., 2007), as well as in learning secondary school mathematics (Reinhold et al., 2020a; b). Prior knowledge in mathematics can therefore be expected to play an important role in the context of learning mathematics from home as well.

Besides these cognitive prerequisites, the mathematics-related motivational and affective orientations introduced in the following represent individual learning preconditions closely associated with academic performance (Reinhold et al., 2021; Hannula et al., 2016). Accordingly, students’ engagement is an established predictor of academic outcomes—also in mathematics (Appleton et al., 2006; Barkatsas et al., 2009; Fung et al., 2018; Skinner et al., 2008). For the purpose of this study, we focus on cognitive engagement (i.e., whether deep learning strategies and adequate cognitive strategies for comprehension are used) and behavioral engagement (i.e., involvement and active participation in classroom and learning activities; Fredricks & McColskey, 2012; Fredricks et al., 2004). How students affectively react to instruction—the “emotional quality of children’s involvement in initiating and carrying out learning activities” (Skinner & Belmont, 1993, p. 572)—can further influence academic performance and learning (Fredricks et al., 2004). If students feel overwhelmed during instruction (i.e., experience excessive demand), their negative evaluations of their own capacity to handle the learning situation might result in lower motivation and engagement, which is in line with expectancy-value theory of achievement motivation (Eccles et al., 1983; Wigfield & Eccles, 2000). Mathematics anxiety (i.e., feeling concerned when working on mathematics problems; Richardson & Suinn, 1972) has a well-documented negative relation to mathematics learning and achievement (Ashcraft & Moore, 2009; Dowker et al., 2016; Hembree, 1990; Ma, 1999)—while mathematics-related self-concept (i.e., students perception about their own mathematical competence; Marsh et al., 2012) is positively related to learning and achievement (Köller et al., 2006; Marsh & Martin, 2011; Möller et al., 2011). Finally, interest in mathematics (i.e., a positive arousal when dealing with mathematics contents) has been shown to increase students’ motivation to engage in mathematics (Pekrun et al., 2006) and to support learning processes (Hidi & Renninger, 2006).

#### External factors: family SES and general teacher support (not pandemic-specific)

Studies focusing on effects of the family SES on learning often analyze consequences of the summer break and inquire after family investments in learning and relate it all to differences in SES. To start with, although there is no agreement on its actual size, research suggests a decline in achievement over the summer break (or, alternatively, at least a flattening of the gain) which is more pronounced for mathematics than for reading. There is evidence for a stronger summer break effect for lower SES as compared to upper SES students (Alexander et al., 2001; Cooper et al., 1996; Quinn et al., 2016). This assumption, however, is challenged by more recent work that controls for measurement artifacts such as changing tests and test scaling. These studies find, at most, small associations of summer achievement loss with poverty or mothers’ education level (Hippel & Hamrock, 2019; Kuhfeld, 2019). Widening the focus on the whole school year, a study based on a nationally representative longitudinal dataset takes a closer look at family investments as one potential driving force behind SES achievement gaps. Initial SES gaps in math and science skills turned out to slightly increase (with small effect sizes between 0.01 and 0.02 *SD* units per 3 months of exposure) in the first 3 years of primary school. While SES skills gaps in science grew during the school year, skills gaps in math intensified during the summer months. Family investments involving home learning activities and out-of-home enrichment activities could partly explain exacerbation of SES performance gaps (Coley et al., 2020). Data from Switzerland and Germany, gathered during the COVID-19 pandemic, indicate reductions in children’s studying time of more than 3 h per day. Neither study, however, found considerable differences in studying time as a function of parental education (Grätz & Lipps, 2021; Wößmann et al., 2020). These data, however, do not preclude the possibility of SES-related differences in the quality of the learning activities during studying time. Despite the controversy on the extent of the influence of SES on students’ quality and quantity of learning from home, we consider family SES as general learning condition to paint a comprehensive picture of the resources available to diverse students—especially since it is particularly the group of students from low-SES families who struggle most in mathematics (Valero et al., 2015).

Regarding the general teacher support provided in regular school contexts (not pandemic-specific), one central assumption in self-determination theory states that it is not the teacher’s behavior per se that influences student motivation and achievement but rather the perception of these behaviors from the student perspective (Spearman & Watt, 2013; Stroet et al., 2013). Just as expectancy-value theory, self-determination theory attaches great importance to subjective (perceived) value as central motive for behavior. In addition, self-determination theory also stresses the influence of situational factors in the process of internalizing extrinsic values. This process is supported if situational factors are perceived as instrumental to the satisfaction of the basic psychological needs of competence, relatedness, and autonomy (Ryan & Deci, 2020). Accordingly, we distinguish between students’ perception of how they are informed about their own competence in mathematics during regular classroom teaching (competence support), students’ perception of how their need for autonomy is addressed during mathematics instruction in school (autonomy support), and students’ perception of their connectedness to other learners in mathematics classes (social relatedness; Deci & Ryan, 2012; Ryan & Deci, 2000). These perceptions influence the way students learn (e.g., Black & Deci, 2000; Ryan & Grolnick, 1986). Focusing on mathematics, autonomy-supportive instruction can have a positive effect on later mathematics motivation and achievement (Froiland et al., 2016; Gutiérrez et al., 2018; Wei et al., 2019). Stroet and colleagues (2013) report in their review article students’ perceptions of needs-supportive instruction to be positively related to their motivation, engagement, and hence achievement.

### The present study

Based on data from 7th-grade secondary school students in Germany gathered via an online survey at the end of the first school year during the COVID-19 pandemic in the summer 2020, we identify systematically occurring groups of students that differ in specific home learning conditions and general learning conditions, comprising internal and external factors related to mathematics learning from home. The resulting student profiles are described by their manifestations on these variables and interpreted as more or less afflicted by extended periods of remote schooling. This study thereby aims at providing insights into available and missing resources and underlying conditions for learning mathematics from home. Due to a lack of existing research on the topic, we decided against formulating exact hypotheses on group sizes and characteristics and, therefore, chose latent profile analysis as the method of analysis, as it allows detecting groups within a dataset without having to define group sizes or characteristics a priori (i.e., a data-driven approach; Gibson, 1959; Hofer & Stern, 2016; and 8; Lazarsfeld & Henry, 1968; Vermunt & Magidson, 2002). We accordingly use the specific home learning internal factors *perceived value* and *success of students’ math learning from home*, the external factors *direct* and *indirect family support*, as well as *teacher support: material* and *teacher support: contact* as profile indicator variables. The general learning conditions *sustained attention*, *math performance*, *engagement*, *excessive demand*, *anxiety*, *interest*, *self-concept* (all internal), *SES* (external), *perceived autonomy support*, *perceived competence support*, and *perceived social relatedness support* (external) are included as outcome variables associated with each profile that further contribute to profile formation. The resulting profile solution is described and interpreted in terms of students’ internal and external resources to handle mathematics learning from home.

## Methods

### Sample and procedure

The sample consists of *N* = 223 7th-grade secondary school students from Germany (*M*_age_ = 12.84 years, *SD*_age_ = 0.55) with *n* = 115 female students, *n* = 106 male students, and *n* = 2 students who indicated that they do not fit in any of those categories. Students were acquired by contacting mathematics teachers from 18 schools in Bavaria, Germany; we were already in contact in the context of another project. We contacted eleven schools from the highest secondary school track (Gymnasium), and eight schools agreed to participate. From the seven lower track secondary schools (Mittelschule) that we contacted, two schools were willing to participate. Schools participated with one to five classrooms, depending on school size. The participating school’s 7th-grade mathematics teachers distributed information material to the students and their parents, and the parents’ written consent was obtained. All students whose parents agreed to participate in the study received a link to our online survey in July 2020 to be completed in the next weeks until the end of the school year as voluntary homework assignment. From March to end of May 2020, students were learning completely from home. Since June 2020, most students were learning partly from home and partly at school. The survey was run via a secure and established tool (Unipark) conforming with the General Data Protection Regulation. The survey could be accessed from both desktop or laptop computers and touch screen devices like tablets or smartphones. The students could click through the survey at their own pace. The last page of the survey was linked to the web-based version of the sustained attention test that is described below in more detail. The whole assessment was intended to be completed in approximately 30 min. After the end of the school year, in August 2020, we closed the survey and obtained the data. Altogether, *N* = 421 people accessed the survey; however, only *N* = 223 students produced valid datasets. The remaining *N* = 198 participants did not finish the survey. The majority stopped in the first third of the survey. Because participation in the survey was not obligatory, the number of students from one class varied considerably—from one student to 23 students at the maximum. Due to the large variation and often small number of students per class, it was not possible to consider the classroom structure in our analyses. Thirty-nine of the students in the final sample attended the lowest and 184 students the highest secondary school track.

### Instruments and scales

Table 1 provides essential information on all instruments and scales used to assess each of the internal and external factors (for more detailed information, please refer to the Online Resource “Instruments and Scales”). The self-report items are explicitly related to mathematics or mathematics instruction and learning, respectively. All questionnaire items in German language as well as a translated English version are available as Online Resource 1. In addition to the central study variables, all students indicated their gender, the school type they attended, and the amount of time they spent on average per week on learning mathematics from home (scale: 1 = no time, 2 = less than 1 h per week, 3 = 1–2 h per week, 4 = 2–3 h per week, 5 = 3–4 h per week, 6 = 4–5 h per week, 7 = more than 5 h per week).

**Table 1:** All instruments and scales used to assess the study variables

Variables		Operationalization	Adapted from	Sample items
Internal factors				
Perceived value and success of mathematics learning from home	Specific home learning condition	4-point Likert scale (1 = almost never, 2 = sometimes, 3 = often, and 4 = almost always) “Perceived value” scale contains three items “Perceived success” scale contains four items	Eccles and Wigfield (1995)	I wanted to get ahead in math even when school was closed Learning math has worked well from home
External factors				
Direct and indirect family support	Specific home learning condition	4-point Likert scale from 1 = almost never to 4 = almost always Students evaluate two statements addressing (1) direct support and (2) indirect support	-	My family supported me in learning math at home When I wanted to work at home for school, I was disturbed
Teacher support: material and contact	Specific home learning condition	4-point Likert scales with 1 = almost never to 4 = almost always “Material” scale: two items capture if and to what extent the math teacher provided material, resources, and structure allowing the students to continue learning math from home “Contact” scale: two items inquire after the extent of contact and the teacher’s presence during remote schooling	-	From my math teacher, I received enough material to study at home My math teacher was in direct contact with me (for example, via Zoom, Skype, chat, WhatsApp, phone calls)
Internal factors				
Sustained attention	General learning condition	For 3 min, students are presented with rows of pictorial flowers that need to be sorted as targets and distractors following discrete rules The employed full cancelation procedure results in an index of sustained attention	Koch et al. (under review)	See Fig. 1 in the appendix “Instruments and Scales”
Math performance	General learning condition	12 items representing basic knowledge of fractions (maximum score = 12) Conceptual knowledge items as well as procedural knowledge items	-	Students are asked to name the fraction depicted in a pie chart with non-equal parts Students are asked to divide 8/35 by 4/15
Engagement	General learning condition	4-point Likert scale from 1 = almost never to 4 = almost always 17 items assess whether deep learning strategies and adequate cognitive strategies for comprehension are used and address involvement and active participation in classroom and learning activities related to mathematics	Wang et al. (2016)	When I don’t understand something in math, I try to clarify it I stay focused in math
Excessive demand	General learning condition	4-point Likert scale from 1 = do not agree at all to 4 = totally agree Three items capture if and to what extent students feel overwhelmed by their math instruction	Prenzel and Drechsel (1996)	In math class, everything goes too fast for me
Anxiety	General learning condition	4-point Likert scale from 1 = do not agree at all to 4 = totally agree fFve items assess mathematics-related anxiety	PISA ANXMAT scale	I worry about getting bad grades in math
Interest	General learning condition	4-point Likert scale from 1 = do not agree at all to 4 = totally agree Three items assess interest in mathematics	Prenzel and Drechsel (1996)	In math class, I’m curious
Self-concept	General learning condition	4-point Likert scale from 1 = do not agree at all to 4 = totally agree Five items assess self-concept in mathematics	PISA SCMAT scale	I have always been convinced that math is one of my best subjects
External factors				
Socioeconomic status (SES)	General learning condition	Students answer two open questions about their parents’ current occupation and the specific work in their profession SES is measured via the International Socio-Economic Index of Occupational Status with values ranging between 16 (cleaners and agricultural assistants) and 90 (judges) Family SES is defined by the highest SES score in the family	Ganzeboom and Treimann (1996)	-
Competence, autonomy, and social relatedness support	General learning condition	4-point Likert scales from 1 = do not agree at all to 4 = totally agree “Perceived competence support”: five items assess the students’ perception of the awareness, communication, and appreciation of competence in their math lessons “Perceived autonomy support”: six items focus on the students’ perception of the degree of autonomy they have in their math lessons “Perceived social relatedness”: five items address students’ perception of the social climate and, in particular, the level of their own integration in the mathematics classroom context	Prenzel and Drechsel (1996)	In math, I am informed about my individual progress In math, I have the opportunity to try out new things myself In math, I feel like I belong

### Statistical analysis

Data compilation and descriptive analyses were conducted in R-4.0.2. We applied latent profile analysis (LPA) to identify groups of students who differ systematically in terms of their internal and external resources to handle the demands of mathematics learning from home. The latent profile analyses were run with the software Mplus Version 8.4 (Muthén & Muthén, 1998-2017) using robust maximum likelihood estimation. The six specific home learning internal and external factors directly associated with learning mathematics from home are included as indicator variables conceptually defining the profiles and guiding their interpretation. The eleven general learning internal and external factors that could be expected to affect the specific home learning conditions are considered outcome variables being associated with each estimated profile. Just as the specific home learning conditions, these general learning conditions accordingly contribute to profile formation. Profiles were hence estimated in one step based on all of the 17 variables (all specific home and general learning conditions). Conceptually, however, the six specific home learning conditions were used as the main descriptors for interpreting the profiles. In order to determine the number of profiles, models with two to five profiles were estimated. Solutions with a higher number of profiles were not only more difficult to interpret but also computationally more complex resulting in less trustworthy solutions given the high number of free parameters. We assessed model fit (i.e., the correspondence between data and the specified latent profile model) for each of the two-to-five-profile solutions by comparing Information Criteria (ICs), looking for the solutions with the lowest (i.e., best) IC values (see, e.g., Geiser, 2011; Gollwitzer, 2012). In line with recommendations of simulation studies, we examined the sample-size adjusted Bayesian Information Criterion (aBIC; Nylund et al., 2007; Sclove, 1987; Tofighi & Enders, 2007; Yang, 2006; Yang & Yang, 2007) and the standard Bayesian Information Criterion (BIC; Schwarz, 1978). In addition, we inspected the Vuong-Lo-Mendell-Rubin (VLMR) likelihood ratio test and the entropy of each profile solution, which indicates the quality of the classification of the students into the estimated profiles. An entropy = 1 would describe perfect, unambiguous classification.

There is no missing data in our final dataset with the exception of two general learning factors: family SES and sustained attention. Twenty-six students did not (reasonably) indicate their parents’ occupation, and *n* = 73 students did not finish the sustained attention test resulting in invalid measures. However, since the proportion of data that was not missing was sufficient for model estimation, we followed the available case approach (pairwise deletion).

## Results

Table 2 provides the means and standard deviations for all study variables as well as the number of items and reliability estimates, whereas all intercorrelations are available in Table 3. There were neither floor nor ceiling effects on any of the variables. Reliability estimates range between 0.62 (for the two-item scale teacher support contact) and 0.92.

**Table 2:** Means, standard deviations, and reliability estimates for the study variables

					Reliability	
Variables		*M*	*SD*	Number of items	*α*	*ω*
Internal						
Perceived success	Specific home learning condition	3.20	0.65	4	.78	.83
Perceived value	Specific home learning condition	2.98	0.75	3	.74	.75
Family support (external)						
Direct family support	Specific home learning condition	2.67	1.04	1	–-	–-
Indirect family support	Specific home learning condition	1.48	0.78	1	–-	–-
Teacher support (external)						
Teacher support material	Specific home learning condition	3.71	0.56	2	.78	–-
Teacher support contact	Specific home learning condition	3.05	0.89	2	.62	–-
Internal						
Sustained attention	General learning condition	8.37	26.40	–-	.86^a^	–-
Math performance	General learning condition	8.65	3.55	12	.89	.91
Engagement	General learning condition	3.05	0.48	17	.87	.90
Excessive demand	General learning condition	2.01	0.68	3	.80	.81
Anxiety	General learning condition	1.97	0.77	5	.89	.92
Interest	General learning condition	2.55	0.71	3	.82	.83
Self-concept	General learning condition	2.62	0.74	5	.88	.91
Family support (external)						
SES	General learning condition	61.59	16.17	–-	–-	–-
Teacher support (external)						
Perceived autonomy support	General learning condition	2.81	0.49	6	.64	.75
Perceived competence support	General learning condition	3.05	0.56	5	.73	.77
Perceived social relatedness	General learning condition	3.04	0.61	5	.80	.84

*Note*. *M* mean, *SD* standard deviation

^a^To estimate the reliability of sustained attention, the split-half reliability has been estimated. To avoid overestimation, this split was carried out by separating the items at the 90 s mark (i.e., after half of the test time has passed)

**Table 3:** Correlations of the study variables (1–6: specific home learning conditions; 7–17: general learning conditions)

Variables	1	2	3	4	5	6	7	8	9	10	11	12	13	14	15	16	17
Internal																	
1. Perceived success	–-																
2. Perceived value	.55	–-															
Family support (external)																	
3. Direct family support	.14	.15	–-														
4. Indirect family support	− .37	− .22	− .09	–-													
Teacher support (external)																	
5. Teacher support material	.18	.24	.01	− .08	–-												
6. Teacher support contact	.01	.13	.05	− .03	.23	–-											
Internal																	
7. Sustained attention	.15	.12	− .05	− .03	.06	− .13	–-										
8. Math performance	.36	.27	.08	− .18	.19	− .10	.57	–-									
9. Engagement	.60	.59	.10	− .31	.23	− .07	.45	.52	–-								
10. Excessive demand	− .54	− .27	− .01	.19	− .12	− .06	− .25	− .31	− .55	–-							
11. Anxiety	− .50	− .18	− .10	.19	− .05	.10	− .16	− .31	− .50	.73	–-						
12. Interest	.32	.48	.10	− .15	.06	.01	.17	.26	.63	− .42	− .32	–-					
13. Self-concept	.45	.23	.03	− .18	.05	− .09	.31	.40	.59	− .68	− .79	.50	–-				
Family support (external)																	
14. SES	.21	.13	.18	− .07	− .06	− .05	.29	.49	.31	− .28	− .24	.19	.35	–-			
Teacher support (external)																	
15. p. autonomy s	.36	.39	− .01	− .07	.32	.09	.16	.29	.47	− .43	− .30	.45	.32	.14	–-		
16. p. competence s	.32	.26	− .03	− .08	.37	.14	.06	.20	.31	− .32	− .23	.28	.24	.09	.50	–-	
17. p. social relatedness	.37	.42	.11	− .13	.28	.05	.25	.36	.42	− .43	− .35	.37	.36	.17	.59	.51	–-

### Identification of profile number

When comparing the solutions with two to five profiles, the four-profile solution fitted the data best (see Table 4). It showed the lowest (i.e., best) BIC, which sanctions complexity more than other information criteria (see Bacci et al., 2014). The aBIC would have suggested the five-profile solution and, secondly, the four-profile solution. The five-profile solution, however, led to problems with the estimation of the standard errors of model parameters (as was the case with all solutions with *k* > 4). Entropies were high (above 0.80) for all profile solutions. The Vuong-Lo-Mendell-Rubin (VLMR) likelihood ratio test indicated the only significant increase in model fit when comparing the one-profile solution to the two-profile solution—emphasizing that a nuanced person-centered perspective may be preferred to a variable-centered approach. In line with Marsh et al. (2009), we also based our decision on the final profile solution on its theoretical meaningfulness, parsimony, and interpretability.

**Table 4:** Logarithmized likelihood (Log L), number of parameters (k), aBIC, BIC, the Vuong-Lo-Mendell-Rubin (VLMR) likelihood ratio test, and the entropy for different LPA solutions

Number of profiles	Log *L*	*k*	aBIC	BIC	VLMR	Entropy
2	− 4887.54	63	9916.07	10,115.72	*p* < 0.001	0.91
3	− 4767.65	92	9741.20	10,032.76	*p* = 0.249	0.89
4	− 4675.05	121	9620.90	10,004.37	*p* = 0.134	0.92
5	− 4599.50	150	9534.71	10,010.08	*p* = 0.157	0.93

Since the final four-profile model fitted the data worse when within-profile correlations between the six indicator variables (i.e., the six specific home learning conditions: perceived success, perceived value, direct family support, indirect family support, teacher support material, teacher support contact) were set free, we could assume to meet the assumption of local independence implying that the indicator variables are uncorrelated within each profile (Lazarsfeld & Henry, 1968). Moreover, model estimation improved with the variance of each indicator variable being constrained to be equal across profiles compared to unrestricted estimation. Consequently, there was no need to relax the homogeneity of variance assumption.

### Description of the final profile solution based on the six specific home learning internal and external factors

**Fig. 2:**
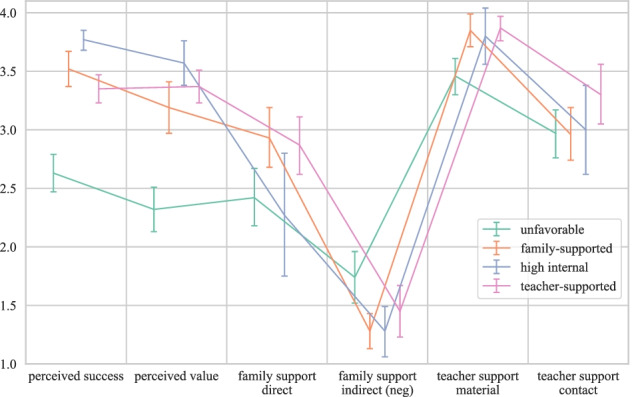
Estimated means on the six specific home learning indicator variables for the four profiles, with error bars representing the 95% confidence intervals

Figure 2 depicts the mean estimated values on the six indicator variables for the four profiles with error bars representing the 95% confidence intervals. These estimates are also provided in Table 5 together with profile-specific membership proportions and counts. Although, in LPA, individuals are not deterministically but rather probabilistically assigned to each profile, group-size specifications are based on the most likely latent profile membership for each student. In the following, we describe each of the profiles along the six specific home learning internal and external factors that directly reflect students’ resources to handle mathematics learning from home. Our analyses are not confirmatory in the sense that we test a specific model or hypothesis. We rather apply an explorative approach to learn more about potential student profiles related to resources for learning from home. We hence conduct significance tests to compare the estimated means between the profiles for our profile indicator variables (the specific home learning conditions, which are conceptually at the center of our profiles), but not for the general learning conditions that were estimated as profile outcomes (see “Profile-specific internal and external general learning factors related to learning mathematics from home” section). All comparisons (i.e., significance tests) between the profile indicators are provided as Online Resource 2 and based on the chi-square value (*χ*2) of the Wald test of parameter constraints.

**Table 5:** Profile-specific membership counts (*n*) and proportions based on the most likely latent profile membership pattern as well as estimates for the six specific home learning conditions for the final four profiles

			Specific home learning conditions					
Profile	*n*	Proportions	Internal		Family support (external)		Teacher support (external)	
			Perceived success [95% CI]	Perceived value [95% CI]	Direct family support [95% CI]	Indirect family support [95% CI]	Teacher support material [95% CI]	Teacher support contact [95% CI]
Unfavorable	77	0.35	2.63 [2.47, 2.79]	2.32 [2.13, 2.51]	2.42 [2.18, 2.67]	1.74 [1.52, 1.96]	3.46 [3.30, 3.61]	2.97 [2.76, 3.17]
Family-supported	67	0.30	3.52 [3.37, 3.67]	3.19 [2.97, 3.41]	2.93 [2.68, 3.19]	1.28 [1.13, 1.43]	3.85 [3.71, 3.99]	2.96 [2.74, 3.19]
High internal	25	0.11	3.77 [3.68, 3.85]	3.57 [3.38, 3.76]	2.27 [1.75, 2.80]	1.28 [1.06, 1.49]	3.80 [3.56, 4.04]	3.00 [2.62, 3.38]
Teacher-supported	54	0.24	3.35 [3.23, 3.47]	3.37 [3.23, 3.51]	2.87 [2.62, 3.11]	1.45 [1.23, 1.67]	3.87 [3.76, 3.97]	3.30 [3.05, 3.56]

*Note*. *CI* confidence interval. The overall standard deviation was *SD* = 0.48 for perceived success, *SD* = 0.57 for perceived value, *SD* = 1.01 for direct family support, *SD* = 0.75 for indirect family support, *SD* = 0.52 for teacher support material, and *SD* = 0.88 for teacher support contact

#### Unfavorable profile

Students in this profile (35% of the sample) show the lowest values of all students in the sample in terms of both internal factors perceived success and perceived value of learning math from home. They only sometimes received direct support from their family and were sometimes disturbed when learning at home for school, which indicated less indirect family support (i.e., more disturbances) than all other students in the sample. These students received regular teacher support in terms of the provision of material but again the least support in the sample. Similar to almost all other profiles, students in this profile reported regular teacher support with a focus on the teacher’s presence during remote schooling. Given the overall rather unfavorable internal and external conditions regarding learning for mathematics from home relative to the rest of the sample, this profile can be described as unfavorable. About 30% of all female and 41% of all male students in the sample could be categorized into this profile. Moreover, 25% of all students from the highest secondary school track and 79% of all students from the lower secondary school track belong to the unfavorable profile. These students, on the average, spend 2–3 h per week on learning mathematics from home (*M* = 3.92, *SE* = 0.15). Based on the BCH method for distal outcomes (Asparouhov & Muthén, 2014; Bakk & Vermunt, 2016), which is implemented in Mplus and applies a corrected three-step approach to include auxiliary variables (i.e., distal outcomes) in the model without affecting the model estimates (e.g., profile sizes and characteristics), the estimate of the average time spent on learning mathematics from home for this profile was significantly smaller than all but the family-supported profile (all *p*s < 0.05).

#### Family-supported profile

The students in this profile (30% of the sample) are characterized by rather high success expectancies with high-to-medium perceived task value compared to the other students in the sample. They show the highest manifestations on direct family support and—together with the next profile—the highest indirect family support (i.e., the least disturbances). Their estimates in terms of both teacher support indicators are inconspicuous. Accordingly, these students stand out by their consistently high family support during learning from home. Almost 34% of the female students and 26% of the male students as well as 36% of the highest secondary school track students and no student from the lower secondary school track were in this profile. Similar to the unfavorable profile, students in this profile also spend approximately 2–3 h per week on learning mathematics from home (*M* = 4.23, *SE* = 0.15).

#### High internal profile

This profile comprises 11% of the sample and is characterized by the highest manifestations on both specific home learning internal factors in the sample. They also indicated the lowest direct family support compared to all other students, however, combined with the highest indirect family support (together with the family-supported profile). With both teacher support indicators showing no striking deviations, these students are referred to as the high internal profile. This profile comprises 6% of all female and 17% of all male students as well as 13% of all highest secondary school track students and 3% of all lower secondary school track students. These students spend around 3–4 h per week with learning mathematics from home (*M* = 4.77, *SE* = 0.29).

#### Teacher-supported profile

Finally, the remaining 24% of the students indicated high-to-medium success expectancies and rather high perceived task value compared to the other students in the sample. They seem to regularly receive direct family support (similar to the family-supported profile) and show rather average manifestations in terms of indirect family support relative to the rest of the students. Just as most of the other profiles (except for the unfavorable profile), these students reported that their teachers almost always provided the material and resources necessary to learn mathematics from home—even with the highest estimate compared to the other profiles. However, this profile particularly stood out by its high manifestation on the second teacher support indicator, inquiring after the presence of the teacher during remote schooling. Due to the consistently high ratings on both teacher support indicators, we label this profile as the teacher-supported profile. About 30% of the female students, 16% of the male students and the two students who indicated being neither male nor female belonged to this profile. This profile further encompasses 26% of the highest secondary school track and 18% of the lower secondary school track students. Students in this profile also reported to spend around 3–4 h per week with learning mathematics from home (*M* = 4.55, *SE* = 0.18).

### Profile-specific internal and external general learning factors related to learning mathematics from home

**Fig. 3:**
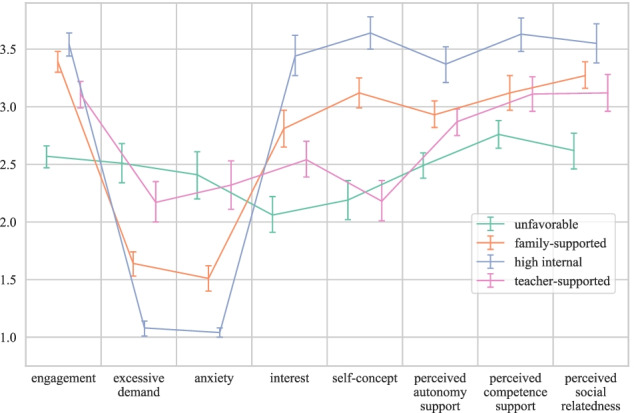
Estimated means on the Likert-scale general learning conditions that were estimated as profile outcomes, with error bars representing the 95% confidence intervals

**Fig. 4:**
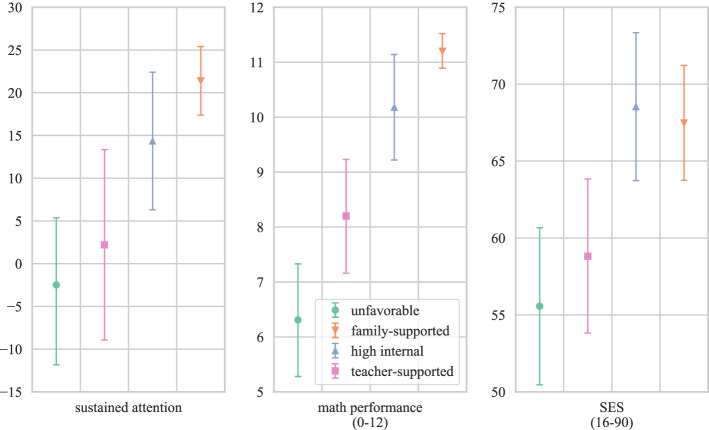
Estimated means on the general learning conditions sustained attention, math performance, and SES that were estimated as profile outcomes with error bars representing the 95% confidence intervals

Table 6 lists the estimated means, standard deviations, and 95% confidence intervals for all general learning factors that were estimated as profile outcomes. Figure 3 and Figure 4 provide the corresponding graphical representation. The profile comparisons reported in the following are based on an inspection of the confidence intervals.

**Table 6:** Means, standard deviations, and 95% confidence intervals for the eleven general learning conditions estimated for each of the final four profiles

General learning conditions	Profile	*M*	*SD*	95% CI
Internal				
Sustained attention	Unfavorable Family-supported High internal Teacher-supported	− 2.48 21.40 14.35 2.20	31.01 12.95 17.10 28.37	[− 11.83, 5.37] [17.38, 25.41] [6.30, 22.41] [− 8.94, 13.33]
Math performance	Unfavorable Family-supported High internal Teacher-supported	6.31 11.20 10.18 8.20	3.96 0.89 2.33 2.95	[5.28, 7.33] [10.89, 11.52] [9.22, 11.14] [7.16, 9.23]
Engagement	Unfavorable Family-supported High internal Teacher-supported	2.57 3.39 3.54 3.11	0.34 0.28 0.23 0.30	[2.47, 2.66] [3.30, 3.48] [3.44, 3.64] [2.99, 3.22]
Excessive demand	Unfavorable Family-supported High internal Teacher-supported	2.51 1.64 1.08 2.17	0.66 0.36 0.14 0.42	[2.34, 2.68] [1.53, 1.74] [1.01, 1.14] [2.00, 2.35]
Anxiety	Unfavorable Family-supported High internal Teacher-supported	2.41 1.51 1.04 2.32	0.79 0.34 0.08 0.56	[2.20, 2.61] [1.40, 1.62] [1.00, 1.08] [2.11, 2.53]
Interest	Unfavorable Family-supported High internal Teacher-supported	2.06 2.81 3.44 2.54	0.57 0.63 0.40 0.52	[1.91, 2.22] [2.65, 2.97] [3.27, 3.62] [2.39, 2.70]
Self-concept	Unfavorable Family-supported High internal Teacher-supported	2.19 3.12 3.64 2.18	0.62 0.39 0.35 0.45	[2.02, 2.36] [2.99, 3.25] [3.50, 3.78] [2.01, 2.36]
Family support (external): SES	Unfavorable Family-supported High internal Teacher-supported	55.57 67.48 68.54 58.82	18.85 10.85 11.22 16.04	[50.46, 60.67] [63.75, 71.22] [63.74, 73.35] [53.82, 63.83]
Teacher support (external)				
Perceived autonomy support	Unfavorable Family-supported High internal Teacher-supported	2.49 2.93 3.37 2.87	0.43 0.41 0.37 0.39	[2.38, 2.60] [2.82, 3.05] [3.21, 3.52] [2.75, 2.98]
Perceived competence support	Unfavorable Family-supported High internal Teacher-supported	2.76 3.12 3.63 3.11	0.52 0.55 0.30 0.44	[2.64, 2.88] [2.97, 3.27] [3.48, 3.77] [2.96, 3.26]
Perceived social relatedness	Unfavorable Family-supported High internal Teacher-supported	2.62 3.27 3.55 3.12	0.61 0.43 0.42 0.49	[2.46, 2.77] [3.16, 3.39] [3.38, 3.72] [2.96, 3.28]

*Note*. *CI* confidence interval

As presented in Table 6, the unfavorable profile indeed shows the lowest manifestations on all general learning internal and external factors except for self-concept in mathematics. This variable was rated slightly lower by students in the teacher-supported profile. Students in the family-supported profile indeed had the highest family SES—together with the high internal profile. The same holds for math performance and sustained attention. In addition to the beneficial manifestations on these two internal factors and family support (SES), the high internal profile also yielded the highest estimates on all remaining general learning internal factors. Interestingly, also their average ratings in terms of perceived competence, autonomy, and social relatedness support turned out to be higher compared to the rest of the sample. The teacher-supported profile, by contrast, showed only average values on these three variables relative to the other students in the sample. While their family SES was comparable to the students in the unfavorable profile, their math performance was higher but still lower than the performance of the students in the other two profiles. In terms of all other general learning internal factors (except for self-concept in mathematics), descriptively, they showed higher manifestations than the unfavorable profile but scored below the other two profiles.

Despite the overall less beneficial general learning conditions, students in the teacher-supported profile did not considerably differ from students in the high internal and family-supported profiles in terms of the specific home learning conditions: They only significantly differed from the high internal profile in terms of perceived success and direct family support and did not significantly differ at all from the family-supported profile. Nevertheless, descriptively, they showed the highest manifestations on teacher support.

## Discussion

In this study, we wanted to find out more about potential differences in the internal and external resources available to students during remote schooling, focusing on secondary school mathematics. We hence identified profiles characterized by students’ perceived success and value of learning from home (specific home learning internal factors) and their families’ as well as their teachers’ support during this time (specific home learning external factors). We related these profiles, which are based on internal and external factors directly associated with learning from home, to more general underlying factors (general learning internal and external factors) such as the student’s cognitive and motivational-affective prerequisites, the family’s SES and the teachers’ competence, autonomy, and social relatedness support during mathematics instruction as perceived by the students. These general learning conditions were intended to further explain the profiles and inform profile interpretation. An adapted version of the Supply-Use-Model (Brühwiler & Blatchford, 2011) was used as an overarching framework to embed our variables (the specific home learning and general learning conditions) and describe their relations. Latent profile analyses suggested a four-profile solution. We labeled the resulting four profiles as the unfavorable, the family-supported, the high internal, and the teacher-supported profile. In the following, we try to synthesize all of the information available to provide a comprehensive interpretation of the four profiles.

### Interpretation of the profiles

Students in three of the four profiles similarly report frequent teacher support in terms of teacher presence and contact. Only students in the teacher-supported profile on average showed higher manifestations. Although teachers were not consistently perceived as “present” during remote schooling, all but the unfavorable profile indicated that their mathematics teachers almost always provided the material and resources necessary to learn from home, representing a slightly more positive picture than existing research that reported rather large variance in the guidance and structure provided by teachers outside the regular school context (Eickelmann & Drossel, 2020; Lohr et al., 2021; Ortiz, 2020; Wößmann et al., 2020). Students in the unfavorable profile, however, also showed significantly lower manifestations on this variable—as well as on all of the three general learning conditions related to teacher support in the mathematics classroom (i.e., competence support, autonomy support, and social relatedness) that have been found to be positively related to students’ motivation, engagement, and hence achievement (e.g., Stroet et al., 2013; Wei et al., 2019). So, they face rather unfavorable external conditions, especially since teaching quality and maintaining close contact seem to be particularly important during remote schooling over and above home resources (Bansak & Starr, 2021; Hofer et al., 2021; Huber & Helm, 2020; Köller et al., 2020).

Regarding direct family support, both the teacher- and the family-supported profiles reported frequent assistance by family members during math learning. A supportive home learning environment—including a dedicated study place at home—can be considered an important protective factor compensating for missing routines and assistance during remote schooling (Andrew et al., 2020; Coley et al., 2020; Köller et al., 2020). Students in the high internal profile might have indicated lower direct family support for math learning because they did not perceive a high need for external support given their high internal resources. This argumentation, however, does not hold for students in the unfavorable profile who also reported less direct family support, combined with the least beneficial manifestations on indirect family support—suggesting a higher frequency of disturbances—compared to the other profiles. These students hence lack at least some of the protective resources that a supportive home learning environment provides (Engzell et al., 2021).

While the unfavorable and the teacher-supported profiles both could be described by a comparably low family SES, the latter profile seems to be equipped with task values related to math learning from home not significantly different from the high internal profile, average success expectancies, and, importantly, high teacher and direct family support. It seems that students in the teacher-supported profile can profit from particularly engaged teachers and a family that provides assistance during math learning from home. Being aware of and encouraged by these external resources might contribute to their expectations of success and the subjective value they assign to math learning from home, which, in turn, have been shown to be related to students’ motivation to engage in (self-regulated) learning (e.g., Artino & Stephens, 2006; Bergey et al., 2018; Eccles & Wigfield, 2002; Kosovich et al., 2017; Panadero & Alonso Tapia, 2014). These resources are not available to students in the unfavorable profile, who accordingly also show the lowest level of engagement and interest in mathematics as compared to all other profiles.

Nearly half of all male students in the sample and more than three quarters of the students from the lower secondary school track belonged to the unfavorable profile. Given that they already demonstrated the lowest mathematics performance in the sample, which is consistent with existing findings relating low SES with more difficulties in mathematics at school (Valero et al., 2015), a prolonged period of intensified learning from home might be particularly harmful for this group of students and considerably increase existing deficits. Although the mere quantity of time students spend with learning mathematics from home is no indicator of the quality or effective learning time (e.g., Romero & Barberà, 2011), students in the unfavorable profile on the average invested significantly less time than all other profiles except for the family-supported profile. While other studies did not find considerable differences in studying time as a function of parental education (Grätz & Lipps, 2021; Wößmann et al., 2020), the person-centered approach used in the present study indicates that low family SES (similar to parental education) can be associated with less studying time during the pandemic if it is accompanied by additional rather detrimental conditions. More generally, our person-centered approach can at least partially explain inconsistent results on the extent of the influence of SES on students’ quality and quantity of learning from home (e.g., Coley et al., 2020; Grätz & Lipps, 2021; Wößmann et al., 2020): Students in the teacher-supported profile, who also showed a comparably low family SES similar to those in the unfavorable profile, nevertheless could rely on a number of protective factors, such as rather high direct family support, teacher support, and task value.

The high internal and the family-supported profile, in turn, are similar in terms of sustained attention and math performance as well as family SES. The high internal profile, however, is characterized by higher manifestations on all other specific home learning and general learning internal factors and on the general learning conditions related to teacher support (i.e., competence support, autonomy support, and social relatedness). The latter might indicate that these variables—being self-reported and referring to the students’ perception of the teacher’s instruction—to a large extent reflect the interaction between students’ internal resources and the teacher’s support during instruction (e.g., Flunger et al., 2019; Seidel, 2007). Two interrelated, additive effects could be expected: Teachers might react differently to motivated, eager, and high-performing students, and these students might perceive math instruction as more satisfying than other students. (The same arguments apply, of course, to the comparatively low expressions of these variables among students in the unfavorable profile—just the other way around.) While there are more female students in the family-supported profile, there are more male students in the high internal profile. Since we focus on mathematics in this study, a male overrepresentation in the high internal profile is well in line with existing research showing a male advantage in terms of motivational-affective variables in the domain of mathematics and STEM more general (e.g., Hofer & Stern, 2016; Reinhold et al., 2019).

Our results complement with the findings by Schult et al. (2021) and Engzell et al. (2021) who reported pronounced negative effects of the pandemic on competence development for students from families with low socio-cultural capital. While we did not assess students’ competence development during the pandemic, this study contributes to a more differentiated understanding of potential detrimental and protective factors accounting for or preserving against learning loss.

### Limitations

A limitation of this study is that all measures are based on information provided by the students. This is inherently consistent as long as internal factors are concerned. With regard to direct and indirect family support and teacher support, measures that do not depend on the student’s perception might be better suited to allow an objective assessment of the situation. However, ultimately, it is all about the students’ handling of the situation. In consequence, their subjective assessment of external factors could also be considered adequate.

Unfortunately, a smaller proportion of lower than higher secondary school students participated in the study. Students attending the two different school types also differ systematically on a number of other variables (e.g., Götz et al., 2013; Sälzer et al., 2013). For instance, both existing research and our own data suggest that students from the lower secondary school track, on the average, come from families with lower SES than students from the highest secondary school track (e.g., Ditton & Maaz, 2011; Stern & Hofer, 2014). A more balanced sample accordingly might have resulted in even more differentiated profiles particularly as regards the description of students from the lower secondary school track. Closely related, there might be a more general selection bias in our sample, since students could opt in or out of the study on a voluntary basis. The students who participated and completed the survey could be assumed to differ systematically from those students not willing to participate or abandoning the survey (e.g., Hernán et al., 2004; Smart, 1966). Since we could expect the self-selection of students into the sample to rather result in disproportionally high manifestations on variables that are positively associated with educational success and perseverance, we might have missed to identify more unfavorable profiles. The profiles described in this study could hence be considered a slightly overoptimistic representation of the actual situation at secondary schools.

### Implications for research

LPA and other person-centered analytical methods constitute fruitful approaches to capture individual differences in cognition, experience, and behavior (e.g., Hickendorff et al., 2017; Hofer et al., 2020). Students’ handling of remote schooling or, more general, learning from home can be assumed to depend on multiple internal and external resources. Different combinations of resources could jointly constitute more or less beneficial conditions for remote schooling with high manifestations on one variable compensating for low manifestations on other variables. This multivariate perspective can be considered particularly valuable when strong unifying effects, for example, of classroom instruction, are absent—as it is the case when learning from home or during the summer break. While recent evidence indicates that demographic factors such as ethnicity or SES account for about four percent of the variability in learning during the summer break, overall, the way how students’ competences develop shows higher variability during the summer months than during the school year (Atteberry & McEachin, 2019; Kuhfeld et al., 2020)—suggesting different internal and external student variables, in addition to SES, to contribute to knowledge development in times without collective classroom instruction.

By contrast, the manifestations of individual variables, inspected in isolation, might be of little value for the prediction of a student’s success to learn mathematics—or any other subject—from home. When trying to describe and understand differences in experience and behavior that are most likely influenced by multiple internal and external variables, an idiosyncratic approach might be preferred over a nomothetic approach.

Future research might also include a performance measure after a period of extended home learning to investigate the association between profile membership and actual learning. Because of considerable variation in the duration and extent of remote schooling and the ratio between classroom instruction and learning at home between schools and grade levels as well as large variation between teachers on a more general level, relating performance differences to differences in profile membership is not trivial. Large-scale national and international assessments that evaluate student performance relative to certain standards and allow for comparisons of cohorts over time, however, could be linked to student data providing information on profile membership.

### Practical implications

Although bearing in mind the slight overrepresentation of students from the highest secondary school track, this study still provides a quite positive perspective on students’ resources in the face of learning mathematics from home. Students often reported values above the scale mean—or below the scale mean for the negatively coded indirect family support, respectively—on the specific home learning internal and external factors directly related to remote schooling. Exceptions are the unfavorable profile in terms of perceived value and direct family support and the high internal profile also in terms of direct family support. The students felt well supported by their mathematics teachers regarding the provision of material and resources to keep studying even when schools were closed. Although the present data does not allow any causal conclusions, an even more intense contact with the teacher via direct or indirect communication as reflected in the teacher-supported profile might also positively affect the students’ perceived value and success of mathematics learning from home, particularly in students from less advantaged families and lower school types.

Whereas most of the student groups identified in the latent profile analysis in this study can be considered to possess rather high resources regarding several of the specific home learning conditions, students in the unfavorable profile might be seriously at risk of losing ground when there is an increased necessity for learning from home. These are the students—who are often male and from the lower secondary school track—we should specifically attend to (see Agasisti & Longobardi, 2017). Interventions targeting the materials as well as the structure and guidance provided by teachers during learning from home (e.g., Begeny et al., 2020), on the one hand, and the home learning environment, on the other hand, could be aimed at this group of particularly vulnerable students (see Elliott & Bachman, 2018). Importantly, educators might assist these students in developing higher internal resources to be more resilient in the face of unfavorable external conditions. Students could hence be supported to improve not only their content knowledge but also their self-regulation and learning strategies. Out-of-school tutoring might be an adequate means here—on condition that families do not have to pay for it (e.g., Heinrich et al., 2014; Rheinheimer et al., 2010).

## Conclusion

When students’ prior knowledge, sustained attention, and motivational-affective preconditions are rather impeding mathematics learning than facilitating it, when they come from lower SES families where direct support during learning and indirect support in the form of a distraction-free workplace are less often available, along with less favorable experiences during regular teacher instruction in mathematics and less teacher support during home learning, students also tend to show considerably lower perceived value and success in the face of mathematics learning from home compared to other students. However, this study also suggests that even when general learning internal conditions and the social background of the family are less favorable, family and/or teacher support could substantially improve students’ learning motivation during home learning.

## Supplementary Information

Below is the link to the electronic supplementary material.

Supplementary file1 (PDF 350 KB)

Supplementary file2 (PDF 163 KB)

Supplementary file3 (PDF 119 KB)

## Data Availability

All rating scales used in this study are available as supplementary material. The data can be obtained by contacting the first author.
