# Supplementary material for: Students home alone—profiles of internal and external conditions associated with mathematics learning from home
Source: Eur J Psychol Educ. 2022 Jan 5;38(1):333–66. doi: 10.1007/s10212-021-00590-w (PMC8727485; doi:10.1007/s10212-021-00590-w)
Supplement: Supplementary file 1 — Supplementary file1 (PDF 350 KB) [file 10212_2021_590_MOESM1_ESM.pdf]

Online Resource "Instruments and Scales"

Students home alone – Profiles of internal and external conditions associated with mathematics learning from home; European Journal of Psychology of Education; Hofer, S. I., Reinhold, F., Koch, M.

1. Internal factors

Perceived value and success of mathematics learning from home. The two home learning internal factors were assessed with two scales inspired by Eccles and Wigfield (1995) and answered on a 4-point Likert scale with 1 = almost never, 2 = sometimes, 3 = often, and 4 = almost always. The "perceived value" scale contains three items including "I wanted to get ahead in math even when school was closed" or "It was important for me to learn math at home". The "perceived success" scale consists of four items such as "Learning math has worked well from home" or "I got distracted when I was supposed to be studying math at home (negatively formulated)".

Sustained attention. The attention swiping task (AST; Koch et al., under review) was developed as a test of visual sustained attention that can be administered on any mobile device. Throughout the test, participants are presented with rows of nine stimuli (pictorial flowers) which are constructed based on two dichotomous dimensions, resulting in four different stimulus categories (see Figure 1 A). The instructions are presented self-paced and the participants are required to remember two stimulus categories (the targets) which they will need to push towards the upper third of the screen. All stimuli which do not meet these criteria (the distractors) must be pushed towards the lower third. To further increase the requirements towards participants' sustained attention, the original test was modified in a way that the background color would switch randomly indicating a rule change (i.e., the direction into which stimuli were to be pushed was inverted). After reading the instructions, participants first received a practice row of nine stimuli without any rule changes. After completing this row, they received a practice row with the rules being switched for all nine stimuli. Finally, participants received a row where the rule switched. During the practice rows, participants received visual feedback on their performance (see Figure 1 B). To provide all participants with comparable prerequisites before starting the real test, each time they committed more than three mistakes per practice row, they would be presented with another practice row. This process was repeated up to three times. Once the real test started, no more

feedback was provided, and participants were required to work on the task for three minutes as fast and conscientiously as possible. To constantly remind participants of the time limit, a progress bar was presented at the upper border of the screen, indicating how much time has passed already. Reactions to each item were collected as one of four categories (i.e., hits, omissions, mistakes, and dismissals). For further analyses, a measure of sustained attention was computed by subtracting the number of mistakes and omissions from the hits. This score was used to correct for correct responses that resulted from inattentive guessing. Due to the novelty of this test instrument, there are no norming samples yet and the resulting score has to be interpreted relative to the study sample.

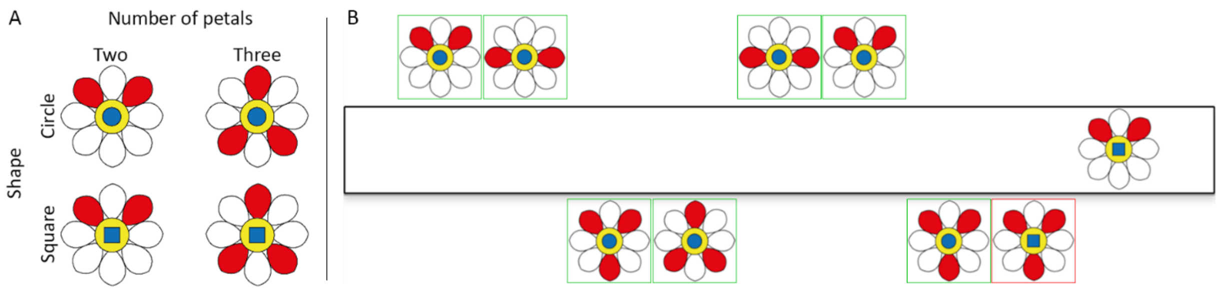

Figure 1. Stimuli and practice row of the AST. In A, all possible combinations of stimuli can be seen. The stimuli vary by the shape in the center and the number of colored petals. For each stimulus, four variants exist to prevent participants from memorizing and thus relying too much on visual comparison. In B, an example from a practice trial is presented. The rule was to move stimuli with a circle and two-colored petals as well as stimuli with a square and three colored petals to the upper part of the screen. The first seven stimuli were categorized correctly and have thus been highlighted with a green box. The eighth stimulus should have also been moved to the upper part; thus, it has been marked red and the participant is required to correct their response before continuing with the last stimulus.

Math performance. With 7th-Graders in focus of the present study, we assessed mathematics performance with 12 items representing basic knowledge of fractions. This content represents the core content of Grade 6 in the present curriculum for all participants—i.e., the last content taught to the students in regular school contexts before the pandemic situation. Basic fraction knowledge is operationalized with both conceptual knowledge items (e.g., students needed to name the fraction

depicted in a pie chart with non-equal parts) as well as procedural knowledge items (e.g., students needed to divide 8/35 by 4/15).

Engagement. The scale originates from a questionnaire by Wang and colleagues (2016). We combined the two subscales "cognitive engagement" and "behavioral engagement", which are highly correlated, within one joint engagement scale. The items assess whether deep learning strategies and adequate cognitive strategies for comprehension are used and address involvement and active participation in classroom and learning activities related to mathematics (17 items). It is based on a 4-point Likert scale from 1 = almost never to 4 = almost always.

Excessive demand. The items used to measure excessive demand are adapted from a scale by Prenzel and Drechsel (1996), apply the 4-point Likert scale from 1 = do not agree at all to 4 = totally agree, and capture if and to what extent students feel overwhelmed by their math instruction (three items). One sample item is "In math class, everything goes too fast for me".

Anxiety. We assessed mathematics-related anxiety with the PISA ANXMAT scale on a 4-point Likert scale from 1 = do not agree at all to 4 = totally agree (five items). The scale consisted of statements like "I worry about getting bad grades in math" (OECD, 2003).

Interest. We adapted three items used by Prenzel and Drechsel (1996) to assess interest in mathematics on a 4-point Likert scale from 1 = do not agree at all to 4 = totally agree. "In math class, I'm curious" is one of the items of this scale.

Self-concept. The PISA SCMAT scale was used to assess students' self-concept in mathematics with a 4-point Likert scale from 1 = do not agree at all to 4 = totally agree (five items). One sample item is "I have always been convinced that math is one of my best subjects" (Marsh et al., 2012).

2. External factors

Direct and indirect family support. To assess whether students directly and indirectly received support from their family during learning at home (home learning factors), we simply asked the students to evaluate (from 1 = almost never to 4 = almost always) the following two statements addressing (1)

direct support and (2) indirect support (negatively formulated): "My family supported me in learning math at home" and "When I wanted to work at home for school, I was disturbed".

Socioeconomic status (SES). Students answered two open questions about their parents' current occupation and the specific work in their profession (Albrecht, 2013), which is considered a valid criterion to determine the socioeconomic status of a family (Baeriswyl et al., 2006). To be able to compare occupations across nations, these answers are transformed to an ISCO-Code (International Standard Classification of Occupations; Albrecht, 2013, p. 3), which is then transferred into a rating scale of professions reflecting the socioeconomic status. One of the most frequently used rating scales is the International Socio-Economic Index of Occupational Status (ISEI; Albrecht, 2013). We also use the ISEI developed by Ganzeboom and Treimann (1996) to indicate SES in this study. This scale has values ranging between 16 (cleaners and agricultural assistants) and 90 (judges; Ganzeboom & Treimann, 1996). There is no consensus whether to report the SES of the mother, of the father or of both parents (e.g., Fertig, 2003). In line with current research integrating information about the SES of both parents (Sirin, 2005), we use the family SES, which is defined by the highest SES score in the family.

Teacher support: material and contact. We constructed the two scales ourselves to assess teacher support. The "material" scale captures if and to what extent the math teacher provided material, resources, and structure allowing the students to continue learning math from home (two items). This scale consists of items like "From my math teacher I received enough material to study at home". The "contact" scale (two items) inquires after the extent of contact and the teacher's presence during remote schooling. One of the items was "My math teacher was in direct contact with me (for example, via Zoom, Skype, chat, WhatsApp, phone calls)". Students answered these items on a 4-point Likert scales with 1 = almost never to 4 = almost always.

Competence, autonomy, and social relatedness support. The following scales were adopted from Prenzel and Drechsel (1996) and only slightly adapted to mathematics instruction. We used 4-point Likert scales from 1 = do not agree at all to 4 = totally agree. The scale "perceived competence support" assesses the students' perception of the awareness, communication, and appreciation of competence in their math lessons (five items). One sample item is "In math, I am informed about my individual

progress”. The scale “perceived autonomy support“ focuses on the students’ perception of the degree of autonomy they have in their math lessons (six items). “In math, I have the opportunity to try out new things myself” is one sample item of this scale. And finally, the scale “perceived social relatedness” addresses students’ perception of the social climate and, in particular, the level of their own integration in the mathematics classroom context (five items). The scale consists of items like “In math, I feel like I belong”.

Albrecht, R. (2013). *International Standard Classification of Occupations (ISCO) & International Socio-Economic Index of Occupational Status (ISEI). Überblick und Übergang zu den 2008er Versionen*. GRIN Verlag GmbH.

Baeriswyl, F., Wandeler, C., Trautwein, U. & Oswald, K. (2006). Leistungstest, Offenheit von Bildungsgängen und obligatorische Beratung der Eltern: Reduziert das Deutschfreiburger Übergangsmodell die Effekte des sozialen Hintergrunds bei Übergangsentscheidungen? *Zeitschrift für Erziehungswissenschaft*, *9*(3), 373–392.

Eccles, J. S., & Wigfield, A. (1995). In the mind of the achiever: The structure of adolescents’ academic achievement related-beliefs and self-perceptions. *Personality and Social Psychology Bulletin*, *21*, 215–225.

Fertig, M. (2003). *Who’s to Blame? The Determinants of German Students’ Achievement in the PISA 2000 Study* (SSRN Scholarly Paper No. ID 392040). Social Science Research Network. Retrieved 2019-08-27, from https://papers.ssrn.com/abstract=392040

Ganzeboom, H. & Treimann, D. (1996). Internationally Comparable Measures of Occupational Status for the 1988 International Standard Classification of Occupations. *Social science research*, *25*, 201–239.

Koch, M., Möller, C., Spinath, F. M. (under review). Are you Swiping, or Just Marking? Development and Evaluation of the Attention Swiping Task.

Marsh, H. W., Xu, M., & Martin, A. J. (2012). *Self-concept: A synergy of theory, method, and application.* In K. R. Harris, S. Graham, T. Urdan, C. B. McCormick, G. M. Sinatra, & J. Sweller (Eds.), *APA educational psychology handbook. Theories, constructs, and critical issues* (Vol 1,

pp. 427–458). American Psychological Association. https://doi.org/10.1037/13273-015

OECD (2003). *The PISA 2003 Assessment framework—mathematics, reading, science and problem solving knowledge and skills*. OECD Publishing.

Prenzel, M., and Drechsel, B. (1996). Ein Jahr kaufmännische Erstausbildung: Veränderungen in Lernmotivation und Interesse [One year of initial business management training: changes in learning motivation and interest]. *Unterrichtswissenschaft*, *24*, 217–234.

Sirin, S. R. (2005). Socioeconomic Status and Academic Achievement: A Meta-Analytic Review of Research. *Review of Educational Research*, *75*(3), 417–453. Retrieved 2019-05-18, from http://journals.sagepub.com/doi/10.3102/00346543075003417

Wang, M. T., Fredricks, J. A., Ye, F., Hofkens, T. L., & Linn, J. S. (2016). The math and science engagement scales: Scale development, validation, and psychometric properties. *Learning and Instruction*, *43*, 16-26.
