# Supplementary material for: Students home alone—profiles of internal and external conditions associated with mathematics learning from home
Source: Eur J Psychol Educ. 2022 Jan 5;38(1):333–66. doi: 10.1007/s10212-021-00590-w (PMC8727485; doi:10.1007/s10212-021-00590-w)
Supplement: Supplementary file 2 — Supplementary file2 (PDF 163 KB) [file 10212_2021_590_MOESM2_ESM.pdf]

Online Resource 1

Students home alone – Profiles of internal and external conditions associated with mathematics learning from home; European Journal of Psychology of Education; Hofer, S. I., Reinhold, F., Koch, M.

Table

Math@Home questionnaire items and scales in English and German language

Variables	English		German	
	Item(s)	Scale	Item(s)	Scale
internal (specific home learning conditions)				
perceived success	I felt overwhelmed learning math at home.	1 = almost never	Ich fühlte mich überfordert, zuhause Mathe zu lernen.	1 = fast nie
	I was able to concentrate on learning math at home.	2 = sometimes	Ich konnte zuhause konzentriert Mathe lernen.	2 = manchmal
	Learning math has worked well from home.	3 = often	Mathe zu lernen hat von zuhause aus gut geklappt.	3 = oft
	I got distracted when I was supposed to be studying math at home.	4 = almost always	Ich ließ mich ablenken, wenn ich zuhause Mathe lernen sollte.	4 = fast immer
perceived value	I wanted to get ahead in math even when school was closed.	1 = almost never	Ich wollte in Mathe weiterkommen, auch wenn die Schule geschlossen war.	1 = fast nie
	It was important for me to learn math at home.	2 = sometimes	Mir war es wichtig, zuhause Mathe zu lernen.	2 = manchmal
	I didn't feel like learning math at home.	3 = often	Ich hatte keine Lust, zuhause Mathe zu lernen.	3 = oft
		4 = almost always		4 = fast immer

(continued)

Table (continued)

Variables	English		German	
	Item(s)	Scale	Item(s)	Scale
internal (general learning conditions)				
engagement	In math, I check if I have understood everything.	1 = almost never	In Mathe kontrolliere ich, ob ich alles verstanden habe.	1 = fast nie
	In math, I know what is important and what is not.	2 = sometimes	Ich weiß in Mathe, was wichtig ist und was nicht.	2 = manchmal
	When I don't understand something in math, I try to clarify it.	3 = often	Wenn ich in Mathe etwas nicht verstehe, versuche ich das zu klären.	3 = oft
	When I learn math, I realize how everything is connected.	4 = almost always	Wenn ich Mathe lerne, ist mir klar, wie alles zusammenhängt.	4 = fast immer
	When I make a mistake in math, I try to understand what I did wrong.		Wenn ich in Mathe einen Fehler mache, versuche ich zu verstehen, was ich falsch gemacht habe.	
	I'd rather be told the answer in math than figure it out myself.		Ich lasse mir in Mathe lieber die Antwort sagen, als selbst eine Lösung zu finden.	
	I turn off my brain when I do something for math.		Ich schalte mein Hirn aus, wenn ich etwas für Mathe mache.	
	When something gets difficult in math, I only deal with the easy parts.		Wenn in Mathe etwas schwierig wird, beschäftige ich mich nur mit den leichten Teilen.	
	I only do as much for math as it is necessary to get through.		Ich tue für Mathe nur so viel wie nötig ist um durchzukommen.	
	I stay focused in math.		In Mathe bleibe ich konzentriert.	
	I put energy into math.		Ich stecke Energie in Mathe.	
	I keep trying even when something in math is difficult.		Ich versuche es weiter, auch wenn etwas in Mathe schwierig ist.	
	I finish my math homework on time.		Ich habe meine Mathehausaufgaben rechtzeitig fertig.	
	I talk about math outside of class.		Ich spreche über Mathe auch außerhalb des Unterrichts.	
	I actively participate in math class.		Ich nehme aktiv am Matheunterricht teil.	
	I do other things when I should be paying attention in math class.		Ich mache andere Dinge, wenn ich im Matheunterricht aufpassen sollte.	
	I give up immediately when I don't understand something in math.		Wenn ich in Mathe etwas nicht verstehe, gebe ich sofort auf.	

(continued)

Table (continued)

Variables	English		German	
	Item(s)	Scale	Item(s)	Scale
excessive demand	In math class, everything goes too fast for me.<br>In math class, the material is too much.<br>The material in math class is too difficult.	1 = do not agree at all<br>2 = rather disagree/ rather not agree<br>3 = rather agree<br>4 = totally agree	Im Matheunterricht geht mir alles zu schnell.<br>Im Matheunterricht ist der Stoff zu viel.<br>Im Matheunterricht ist der Stoff zu schwierig.	1 = stimme überhaupt nicht zu<br>2 = stimme eher nicht zu<br>3 = stimme eher zu<br>4 = stimme völlig zu
anxiety	I often worry that math will be difficult for me.<br>I get very tense when I have to do math problems.<br>I get very nervous when solving math problems.<br>I feel helpless when solving math problems.<br>I worry about getting bad grades in math.	1 = do not agree at all<br>2 = rather disagree/ rather not agree<br>3 = rather agree<br>4 = totally agree	Ich mache mir oft Sorgen, dass es für mich in Mathe schwierig wird.<br>Ich bin sehr angespannt, wenn ich Matheaufgaben machen muss.<br>Beim Lösen von Matheaufgaben werde ich sehr nervös.<br>Ich fühle mich beim Lösen von Matheaufgaben hilflos.<br>Ich mache mir Sorgen, dass ich in Mathe schlechte Noten bekomme.	1 = stimme überhaupt nicht zu<br>2 = stimme eher nicht zu<br>3 = stimme eher zu<br>4 = stimme völlig zu
interest	In math class, I’m curious.<br>I like to deal with math outside of school.<br>In math class I am interested.	1 = do not agree at all<br>2 = rather disagree/ rather not agree<br>3 = rather agree<br>4 = totally agree	Im Matheunterricht bin ich neugierig.<br>Mit Mathe beschäftige ich mich auch außerhalb der Schule gern.<br>Im Matheunterricht bin ich interessiert.	1 = stimme überhaupt nicht zu<br>2 = stimme eher nicht zu<br>3 = stimme eher zu<br>4 = stimme völlig zu

(continued)

Table (continued)

Variables	English		German	
	Item(s)	Scale	Item(s)	Scale
self-concept	I'm just not good at math.<br>I get good grades in math.<br>I learn quickly in math.<br>I have always been convinced that math is one of my best subjects.<br>In math class, I understand even the most difficult problems.	1 = do not agree at all<br>2 = rather disagree/ rather not agree<br>3 = rather agree<br>4 = totally agree	Ich bin einfach nicht gut in Mathe.<br>Im Fach Mathe bekomme ich gute Noten.<br>In Mathe lerne ich schnell.<br>Ich war schon immer überzeugt, dass Mathe eines meiner besten Fächer ist.<br>Im Matheunterricht verstehe ich sogar die schwierigsten Aufgaben.	1 = stimme überhaupt nicht zu<br>2 = stimme eher nicht zu<br>3 = stimme eher zu<br>4 = stimme völlig zu
family support (specific home learning conditions)				
direct family support	My family supported me in learning math at home.	1 = almost never<br>2 = sometimes<br>3 = often<br>4 = almost always	Meine Familie hat mich beim Mathelernen zuhause unterstützt.	1 = fast nie<br>2 = manchmal<br>3 = oft<br>4 = fast immer
indirect family support (negatively coded)	When I wanted to work at home for school, I was disturbed.	1 = almost never<br>2 = sometimes<br>3 = often<br>4 = almost always	Wenn ich zuhause für die Schule arbeiten wollte, wurde ich gestört.	1 = fast nie<br>2 = manchmal<br>3 = oft<br>4 = fast immer

(continued)

Table (continued)

Variables	English		German	
	Item(s)	Scale	Item(s)	Scale
teacher support (specific home learning conditions)				
teacher support material	From my math teacher I received enough material to study at home.<br>My teacher made sure that I could continue learning math from home.	1 = almost never<br>2 = sometimes<br>3 = often<br>4 = almost always	Von meiner/m Lehrer/in hatte ich ausreichend Material zum Lernen für zuhause erhalten.<br>Mein/e Lehrer/in hat dafür gesorgt, dass ich auch von zuhause aus weiter Mathe lernen konnte.	1 = fast nie<br>2 = manchmal<br>3 = oft<br>4 = fast immer
teacher support contact	My teacher checked that I continued to learn math from home (for example, by handing in worksheets, chatting, making phone calls).<br>My teacher was in direct contact with me (for example, via zoom, Skype, chat, WhatsApp, phone calls).	1 = almost never<br>2 = sometimes<br>3 = often<br>4 = almost always	Mein/e Lehrer/in kontrollierte, dass ich auch von zuhause aus weiter Mathe lernte (zum Beispiel durch Abgabe von Arbeitsblättern, Chat, Telefonate).<br>Mein/e Lehrer/in war mit mir in direktem Kontakt (zum Beispiel über zoom, Skype, Chat, WhatsApp, Telefonate).	1 = fast nie<br>2 = manchmal<br>3 = oft<br>4 = fast immer
teacher support (general learning conditions)				
perceived autonomy support	In math, I am allowed to complete tasks in my own way.<br>In math, I am allowed to manage my own time.<br>In math, I am encouraged to work independently.<br>In math, I have the feeling of being strongly controlled.<br>In math, I have the opportunity to work on interesting tasks.<br>In math, I have the opportunity to try out new things myself.	1 = do not agree at all<br>2 = rather disagree/ rather not agree<br>3 = rather agree<br>4 = totally agree	In Mathe darf ich Aufgaben auf meine Art erledigen.<br>In Mathe darf ich mir meine Zeit selbst einteilen.<br>In Mathe werde ich ermutigt, selbstständig zu arbeiten.<br>In Mathe habe ich das Gefühl, stark kontrolliert zu werden.<br>In Mathe habe ich die Gelegenheit, mich mit interessanten Aufgaben zu beschäftigen.<br>In Mathe habe ich die Möglichkeit, neue Dinge selbst auszuprobieren.	1 = stimme überhaupt nicht zu<br>2 = stimme eher nicht zu<br>3 = stimme eher zu<br>4 = stimme völlig zu

(continued)

Table (continued)

Variables	English		German	
	Item(s)	Scale	Item(s)	Scale
perceived competence support	In math, I have enough time to practice.<br>In math, I am praised when I do something well.<br>In math, I am informed about my individual progress.<br>In math, I am told what I can still improve.<br>In math, I am also given credit for difficult tasks.	1 = do not agree at all<br>2 = rather disagree/ rather not agree<br>3 = rather agree<br>4 = totally agree	In Mathe habe ich ausreichend Zeit zu üben.<br>In Mathe werde ich gelobt, wenn ich etwas gut gemacht habe.<br>In Mathe werde ich über meine Fortschritte informiert.<br>In Mathe wird mir gesagt, was ich noch verbessern kann.<br>In Mathe werden mir auch schwierige Aufgaben zugetraut.	1 = stimme überhaupt nicht zu<br>2 = stimme eher nicht zu<br>3 = stimme eher zu<br>4 = stimme völlig zu
perceived social relatedness	My teacher understands me/supports me.<br>In math, I feel that my classmates are responsive to me and understand me.<br>In math, the atmosphere is friendly and relaxed.<br>In math, I feel like I belong.<br>In math, I have the impression that I am taken seriously.	1 = do not agree at all<br>2 = rather disagree/ rather not agree<br>3 = rather agree<br>4 = totally agree	Mein/e Lehrer/in versteht mich/unterstützt mich.<br>In Mathe habe ich das Gefühl, dass meine Mitschüler und Mitschülerinnen auf mich eingehen und mich verstehen.<br>In Mathe ist die Atmosphäre freundschaftlich entspannt.<br>In Mathe habe ich das Gefühl dazuzugehören.<br>In Mathe habe ich den Eindruck, ernst genommen zu werden.	1 = stimme überhaupt nicht zu<br>2 = stimme eher nicht zu<br>3 = stimme eher zu<br>4 = stimme völlig zu

Note. The family support general learning condition socio-economic status (SES) is not mentioned in this table. We assessed the family's SES by means of two open questions about the parents' current occupation and the specific work in their profession.
