# Supplementary material for: Students home alone—profiles of internal and external conditions associated with mathematics learning from home
Source: Eur J Psychol Educ. 2022 Jan 5;38(1):333–66. doi: 10.1007/s10212-021-00590-w (PMC8727485; doi:10.1007/s10212-021-00590-w)
Supplement: Supplementary file 3 — Supplementary file3 (PDF 119 KB) [file 10212_2021_590_MOESM3_ESM.pdf]

Online Resource 2

Students home alone – Profiles of internal and external conditions associated with mathematics learning from home; European Journal of Psychology of Education; Hofer, S. I., Reinhold, F., Koch, M.

Table

Comparisons between the specific home learning conditions estimated for the four profiles

Profile comparisons		internal				family support (external)				teacher support (external)			
		perceived success		perceived value		direct family support		indirect family support		teacher support material		teacher support contact	
		χ^2	p	χ^2	p	χ^2	p	χ^2	p	χ^2	p	χ^2	p
unfavorable	family-supported	63.034	0.000	29.881	0.000	7.757	0.005	11.484	0.001	12.103	0.001	0.000	0.989
	high internal	153.356	0.000	81.866	0.000	0.267	0.606	8.692	0.003	5.183	0.023	0.018	0.893
	teacher-supported	62.048	0.000	77.540	0.000	5.764	0.016	3.189	0.074	18.208	0.000	3.702	0.054
family-supported	high internal	7.620	0.006	6.546	0.011	4.750	0.029	0.000	0.984	0.128	0.720	0.019	0.890
	teacher-supported	3.219	0.073	1.977	0.160	0.135	0.714	1.501	0.221	0.038	0.845	3.532	0.060
high internal	teacher-supported	30.906	0.000	2.766	0.096	3.961	0.047	1.206	0.272	0.258	0.612	1.745	0.187

Note. All comparisons are based on the chi-square value (χ^2) of the Wald test of parameter constraints.
